# Impact of the Fibrosis-4 Index on Risk Stratification of Cardiovascular Events and Mortality in Patients with Atrial Fibrillation: Findings from a Japanese Multicenter Registry

**DOI:** 10.3390/jcm9020584

**Published:** 2020-02-21

**Authors:** Yuki Saito, Yasuo Okumura, Koichi Nagashima, Daisuke Fukamachi, Katsuaki Yokoyama, Naoya Matsumoto, Eizo Tachibana, Keiichiro Kuronuma, Koji Oiwa, Michiaki Matsumoto, Toshihiko Nishida, Toshiaki Kojima, Shoji Hanada, Kazumiki Nomoto, Kazumasa Sonoda, Ken Arima, Fumiyuki Takahashi, Tomobumi Kotani, Kimie Ohkubo, Seiji Fukushima, Satoru Itou, Kunio Kondo, Hideyuki Ando, Yasumi Ohno, Motoyuki Onikura, Atsushi Hirayama

**Affiliations:** 1 Division of Cardiology, Nihon University Itabashi Hospital, 30-1 Ohyaguchi-kamicho, Itabashi-ku, Tokyo 173-8610, Japan; 2 Department of Cardiology, Nihon University Hospital, 30-1 Ohyaguchi-kamicho, Itabashi-ku, Tokyo 173-8610, Japan; 3 Kawaguchi Municipal Medical Center, 180 Nishiaraijuku, Kawaguchi, Saitama 333-0833, Japan; 4 Yokohama Chuo Hospital, 268 Yamashitacho, Naka-ku, Yokohama, Kanagawa 231-0023, Japan; 5 Sekishindo Hospital, 25-19 Wakitahoncho, Kawagoe, Saitama 350-1123, Japan; 6 Asaka Medical Center, 1340-1 Mizonuma, Asaka, Saitama 351-0000, Japan; 7 Tokyo Rinkai Hospital, 1-4-2 Rinkai-cho, Edogawa-ku, Tokyo 134-0086, Japan; 8 Kasukabe Municipal Hospital, 6-7-1 Chuo, Kasukabe, Saitama 344-0067, Japan; 9 Yasuda Hospital, 1-13-9 Narimasu, Itabashi-ku, Tokyo 175-0094, Japan; 10 Makita General Hospital, 1-34-6 Omorikita, Ota-ku, Tokyo 143-0016, Japan; 11 Itabashi Medical Association Hospital, 3-12-6 Takashimadaira, Itabashi-ku, Tokyo 175-0082, Japan; 12 Ukima Central Hospital, 2-21-19 Akabanekita, Kita-ku, Tokyo 115-0052, Japan; 13 Itou Cardiovascular Clinic, 2-4 Higashisumiyoshi, Tokorozawa, Saitama 359-1124, Japan; 14 Kondo Clinic, 2-36-24 Shimoigusa, Suginami-ku, Tokyo 167-0022, Japan; 15 Keiai Clinic, 3-10-23 Mukaihara, Itabashi-ku, Tokyo 173-0036, Japan; 16 Ohno Medical Clinic, 1-36-1 Itabashi, Itabashi-ku, Tokyo 173-0004, Japan; 17 Onikura Clinic, 1-26-13 Katsutadai, Yachiyo, Chiba, 276-0023, Japan

**Keywords:** adverse clinical events, liver fibrosis, Fibrosis-4 index, atrial fibrillation

## Abstract

Background: Liver diseases drive the development and progression of atrial fibrillation (AF). The Fibrosis-4 (FIB4) index is a non-invasive scoring method for detecting liver fibrosis, but the prognostic impact of using it for AF patients is still unknown. Herein, we evaluated using the FIB4 index as a risk assessment tool for cardiovascular events and mortality in patients with AF. Methods: We performed a post-hoc analysis of a prospective, observational multicenter study. A total of 3067 patients enrolled in a multicenter Japanese registry were grouped as first tertile (FIB4 index < 1.75, *n* = 1022), second tertile (1.75 ≤ FIB4 index < 2.51, *n* = 1022), and third tertile (FIB4 index ≥ 2.51, *n* = 1023). Results: The third tertile had statistically significant results: older age, lower body mass index, increased heart failure prevalence, and lower clearances of hemoglobin and creatinine (all *p* < 0.05). During the follow-up period, incidences of major bleeding, cardiovascular events, and all-cause mortality were significantly higher for the third tertile (all *p* < 0.05). After multivariate adjustment, the third tertile associated independently with cardiovascular events (HR 1.72; 95% CI 1.31–2.25) and all-cause mortality (HR 1.43; 95% CI 1.06–1.95). Adding the FIB4 index to a baseline model with CHA_2_DS_2_-VASc score improved the prediction of cardiovascular events and all-cause mortality, as shown by the significant increase in the C-statistic (all *p* < 0.05), net reclassification improvement (all *p* < 0.001), and integrated discrimination improvement (all *p* < 0.001). A FIB4 index ≥ 2.51 most strongly associated with cardiovascular events and all-cause mortality in AF patients with high CHADS_2_ scores (all *p* < 0.001). Conclusions: The FIB4 index is independently associated with risks of cardiovascular events and all-cause mortality in AF patients.

## 1. Introduction

Atrial fibrillation (AF) is currently the most common cardiac arrhythmia, and its prevalence is increasing [1,2]. AF is a strong risk factor for stroke, heart failure, and death [3]. More than 460,000 patient hospitalizations and nearly 80,000 deaths occur annually due to AF in the USA [4]. Since the burden of AF on the healthcare system continues to rise steadily, it has become essential for clinicians to identify AF patients who are at high risk for poor outcomes.

Emerging evidence indicates that liver diseases are closely associated with the development of AF in patients. Studies have reported that the increased incidence rates of AF are linked to liver cirrhosis [5,6], and that non-alcoholic fatty liver disease (NAFLD) is a risk factor for developing AF [7]. Liver diseases cause inflammation that leads to autonomic dysfunction, which produces proarrhythmic substrates leading to the development of AF [8,9]. Despite emerging evidence for the relationship between liver diseases and the progression of AF, it is still very difficult to determine the severity of liver diseases since routine liver tests may be insufficient to diagnose advanced liver fibrosis caused by liver diseases [10].

The Fibrosis-4 (FIB4) index is a simple, non-invasive scoring method for the detection of liver impairment and liver fibrosis [11]. In particular, this method was validated to evaluate liver fibrosis in patients with liver diseases of various etiologies, which include NAFLD, chronic hepatitis C virus (HCV) infection, and HIV/HCV coinfection [11,12,13]. A high FIB4 index is associated with all-cause mortality in several chronic diseases, such as microscopic polyangiitis, rheumatoid arthritis, and heart failure [14,15,16]. However, the prognostic impact of using the FIB4 index for patients with AF is not well understood yet. Therefore, we hypothesized that the FIB4 index is associated with an adverse prognosis in AF patients. In this study, we examined the potential use of the FIB4 index as a risk assessment tool for both cardiovascular events and mortality in patients with AF. Meanwhile, others recommend using the CHADS_2_ score for the assessment of thromboembolic risk in AF patients [17]. This score, which comprises a clustering of risk factors that are associated with increased cardiovascular risk, is used to predict more than just stroke risk assessments. The CHADS_2_ score also predicts cardiovascular events and mortality [18]. However, the risk factors for liver impairment are not considered when calculating the CHADS_2_ score. Therefore, we also investigated whether the FIB4 index would yield predictive values for AF patients that have either low or high CHADS_2_ scores.

## 2. Methods

### 2.1. Study Population

This study was conducted as a post-hoc analysis of a prospective, observational multicenter study from the SAKURA AF Registry (UMIN 000014420) [19,20]. The study design, data collection, and clinical characteristics of the patients were reported previously [19,20]. A total of 3268 patients with non-valvular AF were enrolled in the Registry from September 1st, 2013 to December 31st, 2015. These patients had at least 2 years of follow-up examinations that ended on December 31st, 2017. The patients were enrolled at 63 Tokyo institutions: 2 cardiovascular centers, 13 affiliated hospitals or community hospitals, and 48 private clinics. This study was approved by our institutional review board (IRB) and by individual hospital IRBs. All enrollees provided written informed consent for inclusion in the registry.

### 2.2. Data Collection and Outcome Variables

We used a web-based registration system, which was accessed through the SAKURA AF Registry website, to collect information on de-identified patient clinical characteristics, including comorbidities, medications, and laboratory data. Using a central registry office twice a year in March and September, we obtained patient follow-up data that included international normalized ratios (INRs) for warfarin users, creatinine levels, and hemoglobin levels [19,20]. We also collected data on the initiation of oral anticoagulant (OAC) therapy within 3 months before the registry enrollment of patients. The time in a therapeutic range (TTR) was calculated by the Rosendaal method [21]. We calculated the TTR assuming a PT-INR of 1.6–2.6 for those aged ≥70 years and a PT-INR of 2.0–3.0 for those aged <70 years, according to the 2013 Japanese Circulation Society guidelines [1]. Good PT-INR control was defined arbitrarily as a TTR ≥ 65%. The creatinine clearance (CrCl) was estimated by the Cockcroft–Gault formula.

Four primary outcomes were assessed in this study: strokes, major bleeding, cardiovascular events, and all-cause mortality. Stroke outcomes included ischemic stroke, hemorrhagic stroke, and transient ischemic attack (TIA). Major bleeding was defined as hemoglobin reductions of at least 2 g/dL, the transfusion of at least 2 units of blood, or symptomatic bleeding in a critical area or organ. Cardiovascular events included heart failure, myocardial infarction, unstable angina, and cardiac death.

The CHADS_2_ score was calculated for each patient, in which 1 point was given for each of these criteria that were met: 75-years of age or older, congestive heart failure, hypertension, and diabetes mellitus; and 2 points were given for each of these criteria that were met: previous stroke or TIA [17]. We defined a CHADS_2_ score of ≤1 as a low CHADS_2_ score, and a CHADS_2_ score of ≥2 was defined as a high CHADS_2_ score [22].

### 2.3. Fibrosis-4 index

The FIB4 index was calculated for each patient during enrollment using the following formula [11,12]:

$$FIB4\;Index=\frac{Age(years)\times [Aspartate\;Aminotransferase\left(AST\right)\left(IU/L\right)]}{Platelet\;Count(10^{9}/L)\times \sqrt{Alanine\;Aminotransferase\left(ALT\right)(IU/L)}}$$

Patients who lacked registry data for one or more of the FIB4 index components (aspartate aminotransferase (AST), alanine aminotransferase (ALT), and platelet count) were excluded from this study. We then classified the patients into three groups according to FIB4 index tertiles.

### 2.4. Statistical Analysis

Continuous variables were represented as the mean ± SD and categorical variables as the number (percentage) of patients. The statistical significance for the differences among the continuous variables of the three groups was calculated by using both the one-way analysis of variance (ANOVA) and the Kruskal–Wallis tests. The Chi-squared test analyzed statistical significance for the differences in the categorical variables among these groups. Kaplan–Meier curves were plotted for the cumulative incidences of events, and the log-rank test compared group differences. Using multivariate Cox regression analysis, we analyzed the relationship between the third tertile of the FIB4 index with event incidences. We made analysis adjustments for all components of the CHA2DS2-VASc score (congestive heart failure, hypertension, age ≥ 75 years, diabetes mellitus, vascular disease, and history of stroke or TIA) and other clinically important covariates. 

To assess whether the accuracy of predicting adverse event incidences would improve after adding FIB4 index (continuous variable) to a baseline model with CHA_2_DS_2_-VASc score, the C-statistics, net reclassification improvement (NRI), and integrated discrimination improvement (IDI) were calculated. All statistical analyses were performed using the JMP ver.13.0 software program (SAS Institute, Cary, NC, USA) and the R Statistics version 3.3.1 (R Foundation for Statistical Computing, Vienna, Austria). For all analyses, *p* values less than 0.05 were considered to be statistically significant.

## 3. Results

### 3.1. Patient Characteristics

**Figure 1:**
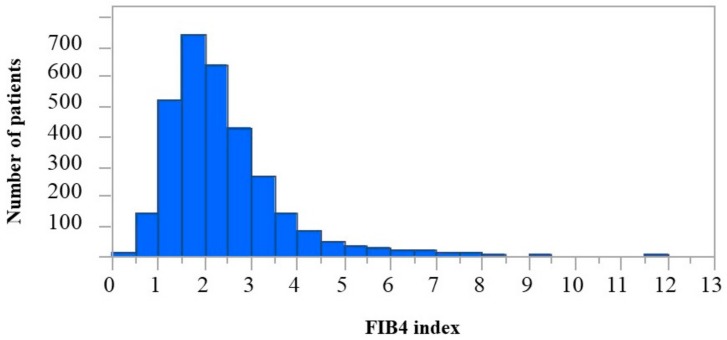
The distribution of the Fibrosis-4 index.

Of the 3268 patients enrolled in the SAKURA AF Registry, we excluded 31 patients who did not meet the follow-up criteria and another 170 patients that lacked data for one or more of the FIB4 index components (AST, ALT, and platelet count). Thus, we analyzed the data sets from a total of 3067 patients. The median (IQR) FIB4 index score was 2.09 (1.59–2.80). The distribution of FIB4 index score is illustrated in Figure 1. The cutoff values for the FIB4 indexes between tertiles were determined to be 1.75 and 2.51, and patients were stratified into three groups: first tertile (FIB4 index < 1.75, *n* = 1022), second tertile (1.75 ≤ FIB4 index < 2.51, *n* = 1022), and third tertile (FIB4 index ≥ 2.51, *n* = 1023). The patient clinical characteristics for these groups are shown in Table 1. When compared to the other two groups, the third tertile group exhibited significance in the following: older age; lower body mass index (BMI); higher prevalence of heart failure (all *p* < 0.05); higher prevalence of both long-standing and persistent AF (*p* < 0.001); higher scores for CHADS_2_, CHA_2_DS_2_-VASc, and HAS-BLED (all *p* < 0.001); and lower counts of hemoglobin, platelets, and CrCl (all *p* < 0.001). For 1321 (90.5%) of 1459 warfarin users, the TTR was available; mean TTR was 65.3 ± 31.0%. Of these, 1321 warfarin users, 765 (57.9%) had good anticoagulation control, defined as TTR >65%.

### 3.2. Clinical Outcomes

**Figure 2:**
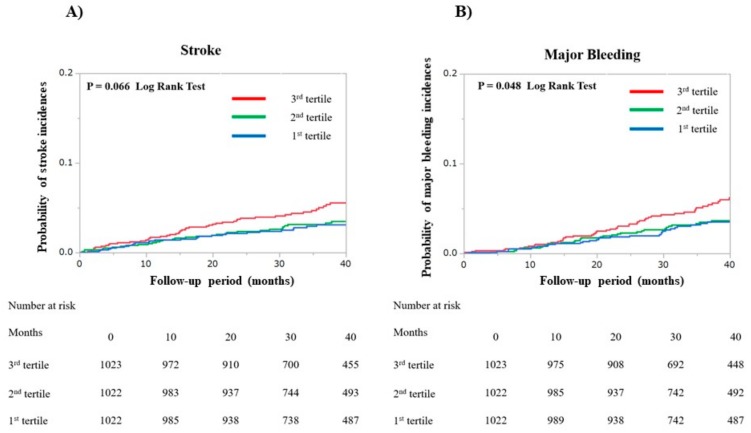
Kaplan–Meier curves were plotted to compare the incidences of both (**A**) strokes and (**B**) major bleeding events for the three patient groups that comprise the first, second, and third tertiles of the Fibrosis-4 index.

**Figure 3:**
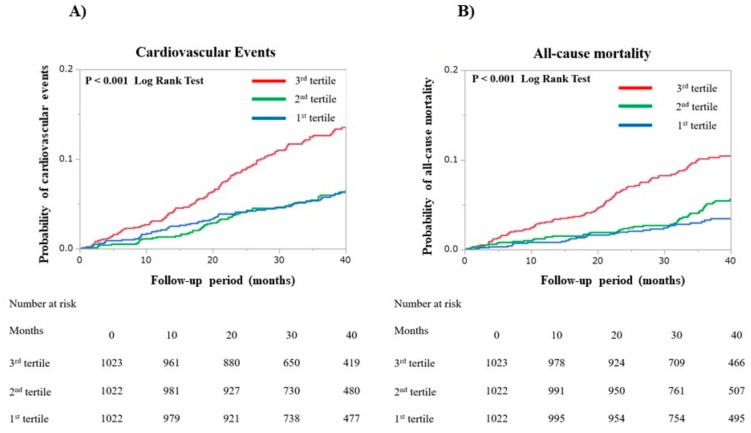
Kaplan—Meier curves were plotted to compare the incidences of both (**A**) cardiovascular events and (**B**) all-cause mortality events for the three patient groups that comprise the first, second, and third tertiles of the Fibrosis-4 index. Cardiovascular events included heart failure, myocardial infarction, unstable angina, and cardiac death.

During the median follow-up period of 39.2 (28.4–43.6) months, the number of patients that experienced the following medical events was: 118 (3.8%) strokes, 119 (3.8%) major bleeding episodes, 248 (8.0%) cardiovascular events, and 190 (6.1%) died. Kaplan–Meier curves for strokes, major bleeding, cardiovascular events, and all-cause mortality are shown in Figure 2 and Figure 3. There were no between-group differences for the incidences of stroke (*p* = 0.066 by log-rank test). The incidence of a major bleeding event was higher for the third tertile group than for either the first or second tertile groups (*p* = 0.048 by log-rank test). The third tertile group also had significantly higher incidences of cardiovascular events and all-cause mortality (all *p* < 0.001 by log-rank test) than the other two groups.

We adjusted all components of the CHA2DS2-VASc score and other covariates related to clinical events and a history of strokes that included TIA. Thus, both the new use of an OAC and a CrCl of ≤ 50 mL/min were independently associated with incidences of stroke. Additionally, a CrCl of ≤ 50 mL/min was independently associated with major bleeding events (Table 2).

After similar adjustments were made to the analysis of the CHA_2_DS_2_-VASc score, the third tertile of the FIB4 index (FIB4 index ≥ 2.51) was independently associated with cardiovascular events (adjusted HR 1.72; 95% CI 1.31–2.25) and all-cause mortality (adjusted HR 1.43; 95% CI 1.06–1.95) (Table 3). The other major determinants for cardiovascular events were: the AF type, history of heart failure, history of vascular disease, new use of an OAC, and CrCl ≤ 50 mL/min; those for all-cause mortality were: age ≥ 75 years, male, bodyweight ≤ 50 kg, history of heart failure, and CrCl ≤ 50 mL/min (Table 3).

### 3.3. Risk Discriminative Power of FIB4 Index

We also investigated whether adding the FIB4 index (continuous variable) to CHA_2_DS_2_-VASc score improved the prediction of cardiovascular events and all-cause mortality. Adding the FIB4 index to a baseline model with CHA_2_DS_2_-VASc score improved the prediction of cardiovascular events (*p* = 0.025) and all-cause mortality (*p* = 0.001), as shown by the significant increase in the C-statistics (Table 4). Reclassification of patients was performed using the NRI and IDI. The NRIs and IDIs for cardiovascular events and all-cause mortality were significantly increased after adding the FIB4 index to a baseline model with CHA_2_DS_2_-VASc score (all *p* < 0.001, Table 4).

### 3.4. Prognostic Role of FIB4 Index in Patients with Either Low or High CHADS_2_ Scores

**Figure 4:**
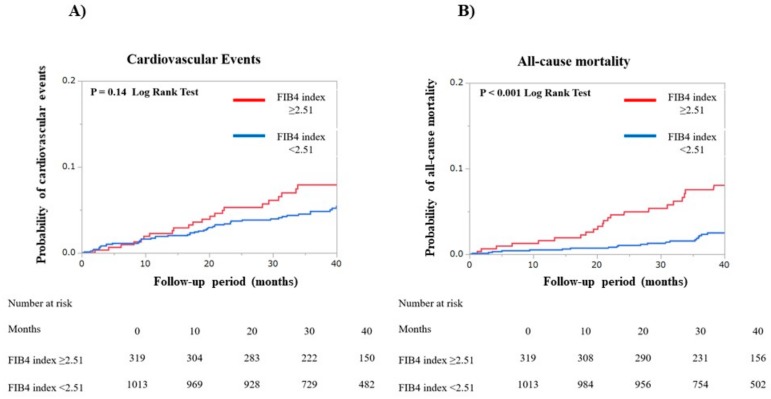
Kaplan—Meier curves were plotted to compare the incidences for both (**A**) cardiovascular events and (**B**) all-cause mortality in two patient groups, according to the Fibrosis-4 index of 2.51 in patients with low CHADS_2_ scores (≤1). Cardiovascular events included heart failure, myocardial infarction, unstable angina, and cardiac death.

**Figure 5:**
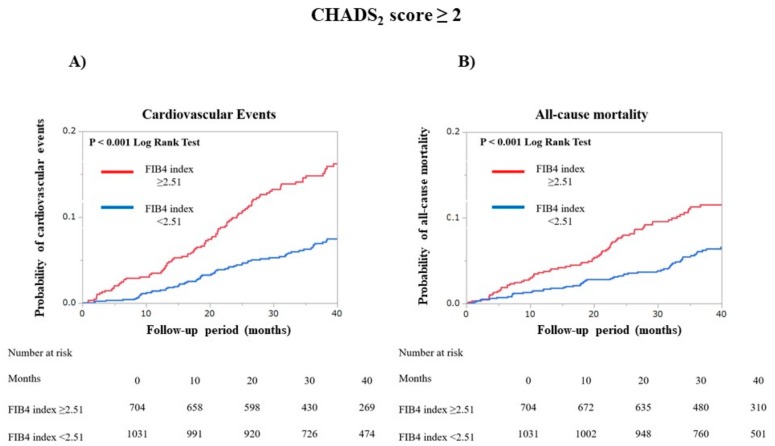
Kaplan—Meier curves were plotted to compare the incidences for both (**A**) cardiovascular events and (**B**) all-cause mortality in two patient groups, according to the Fibrosis-4 index of 2.51 in patients with high CHADS_2_ scores (≥2). Cardiovascular events included heart failure, myocardial infarction, unstable angina, and cardiac death.

We grouped the patients into two groups: a low CHADS_2_ score group (*n* = 1332) and a high CHADS_2_ score group (*n* = 1735). Among the patients with low CHADS_2_ scores, the incidence rate for cardiovascular events did not differ significantly (*p* = 0.14 by log-rank test), but all-cause mortality was significantly higher for those patients with a FIB4 index ≥ 2.51 than for those with a FIB4 index < 2.51 (*p* < 0.001 by log-rank test, Figure 4). For the patients with high CHADS_2_ scores, the incidence rates for both cardiovascular events and all-cause mortality were significantly higher for those with FIB4 indexes of ≥2.51 than for those with FIB4 index < 2.51 (all *p* < 0.001 by log-rank test, Figure 5).

## 4. Discussion

This study is the first to evaluate the association between adverse cardiovascular outcomes in patients with AF and the FIB4 index, which is a non-invasive scoring method for the identification of liver fibrosis. Herein, we presented three key findings. First, AF patients with a high FIB4 index are more likely to have heart failure, anemia, renal dysfunction, and advanced AF, and these patients also had high CHADS_2_, CHA_2_DS_2_-VASc, and HAS-BLED scores. Second, the FIB4 index is independently associated with an increased risk for both cardiovascular events and all-cause mortality in AF patients. Third, adding the FIB4 index to CHA_2_DS_2_-VASc score improved the prediction of cardiovascular events and all-cause mortality. Fourth, the FIB4 index is strongly associated with an increased risk for both cardiovascular events and all-cause mortality, especially in AF patients with high CHADS_2_ scores.

One recent clinical research, including 2330 AF patients, investigated the association between liver fibrosis assessed by FIB4 index and major bleeding events [23]. They reported that FIB4 index > 3.25 was associated with an increased risk of major bleeding events in AF patients on treatment with vitamin K antagonists (VKAs), which was not evident in patients on direct oral anticoagulant (DOAC). The significant association between FIB4 index > 3.25 and cardiovascular events was not founded in that study.

Although it is reported that FIB4 index > 3.25 was validated to predict liver fibrosis in patients with HIV/HCV-coinfected patients [11], the cut-off value of this index could differ among the type of disease. For example, in NAFLD, FIB4 index > 3.25 had a low positive predictive value for prediction of liver fibrosis [24]. Because FIB4 index is calculated by non-specific markers of liver fibrosis, it is not clear this cut-off value could be applied to general population of AF patients. Therefore, in the present study, we divided patients into three groups according to the tertiles and also evaluated the prognostic relevance of the FIB4 index as continuous variable. Our results showed that the third tertile of the FIB4 index (FIB4 index ≥ 2.51) was independently associated with cardiovascular events and all-cause mortality. In addition, adding the FIB4 index (continuous variable) to CHA_2_DS_2_-VASc score improved the prediction of cardiovascular events and all-cause mortality. In our data, the patients with FIB4 index ≥ 2.51 had significantly higher major bleeding incidences, as consistent with previous report [23]. However, FIB4 index ≥ 2.51 was not significantly associated with major bleeding events in multivariate analysis. This may be because our cohort included patients on treatment with both VKAs and DOAC.

Compared to the first tertile group, the second tertile group had older age; lower BMI; higher prevalence of heart failure; higher scores for CHADS_2_, CHA_2_DS_2_-VASc, and HAS-BLED; and lower counts of hemoglobin, platelets, and CrCl. However, the incidences of adverse outcomes were not significantly different among the two groups.

In this study, we found that the FIB4 index is an independent predictor of cardiovascular events and all-cause mortality in patients with AF. Clinical factors such as renal dysfunction, age, heart failure, and coronary artery disease are commonly thought to have negative impacts on the survival of AF patients, but the severity of liver fibrosis has not been viewed as one of these factors [25]. Our results showed that the FIB4 index is independently associated with increased risks for both cardiovascular events and all-cause mortality in AF patients after adjusting for common prognostic clinical factors. Recently, there has been an increasing number of AF patients with multiple comorbidities, such as hypertension, diabetes mellitus, obesity, hyperlipidemia, and metabolic syndrome. Thus, the comprehensive management of overall risk factors, which include liver dysfunction, is necessary to help improve the prognosis of patients with AF (see also Appendix A).

Many studies have shown that advanced stages of AF are associated with adverse cardiovascular outcomes in AF patients [26]. Both persistent and long-standing persistent AF rather than paroxysmal AF are reported to be associated with higher incidences of HF and mortality [26]. Our results showed that the third tertile FIB4 group had a significantly higher prevalence of both persistent and long-standing persistent AF; this group also exhibited significantly lower rates of AF ablation. Thus, the third tertile FIB4 group has more complex AF burdens than the other FIB4 groups. However, the third tertile of the FIB4 index (FIB4 index ≥ 2.51) was independently associated with cardiovascular events and all-cause mortality even after the adjustment for either persistent or long-standing persistent AF versus paroxysmal AF. Therefore, we determined that the FIB4 index is an independent prognostic indicator for identifying the type of AF burden that patients will develop.

Currently, the CHADS_2_ score is widely used for stroke risk stratification in patients with nonvalvular AF [18]. Studies have shown that the CHADS_2_ score also predicts mortality and other cardiovascular events; therefore, the CHADS_2_ score may be used as a quick scoring method to create patient risk-profiles that predict outcomes beyond thromboembolic risk in patients with nonvalvular AF [18]. Our data suggest that the FIB4 index could also be useful for additional risk stratifications for AF patients with high CHADS_2_ scores.

The FIB4 index is also important for several other reasons. First, calculating the FIB4 index is quick and straightforward because the standard examination of any liver disease already includes acquiring the constitutive parameters (age, AST, ALT, and platelet count). Second, the results for the FIB4 index are available immediately, and that allows for it to be used even in time-sensitive emergency cases. Third, the FIB4 index is inexpensive to use. Our data indicate that the FIB4 index provides useful information for predicting future cardiovascular events and mortality in AF patients, and this finding may have important clinical implications for the long-term management of AF patients. Evaluation of FIB4 index is beneficial especially in AF patients with high risk of adverse outcomes (a CHADS_2_ score of ≥2).

However, this study also has some limitations. First, the registry only covered a limited geographical area despite the large-scale prospective study design. Second, the FIB4 index is widely used to assess liver diseases, such as NAFLD and HCV infection, but we do not know if this approach would be suitable to assess the severity of liver injuries in AF patients. Currently, the histological analysis of liver biopsies is the best approach to evaluate liver fibrosis, but a liver biopsy is a very invasive procedure for patients to undergo. Unfortunately, histological samples could not be obtained from AF patients who also had liver comorbidities in our study. Many reports have identified the FIB4 index as a reliable tool to stage liver fibrosis and that the FIB4 index is associated with biochemical markers of liver fibrosis for heart failure patients [12,16]. Therefore, it appears that the prognostic importance of using the FIB4 index in this study was mainly for evaluating liver fibrosis and liver impairment in AF patients. Within the above limitations, our results provide insight into the clinical outcomes for AF patients that are closely associated with the severity of liver injuries.

## 5. Conclusions

Our findings indicate that the FIB4 index, a non-invasive scoring method for evaluating liver fibrosis, is independently associated with the risks of cardiovascular events and all-cause mortality in patients with nonvalvular AF. Therefore, the FIB4 index may be useful as a risk assessment tool for identifying adverse outcomes in patients with AF.

## Figures and Tables

**Table 1:** Clinical characteristics of patients that were stratified into three groups according to tertiles of the Fibrosis-4 index.

Item	First Tertile FIB4 < 1.75 (*n* = 1022)	Second Tertile 1.75 < FIB4 < 2.51 (*n* = 1022)	Third Tertile FIB4 ≥ 2.51 (*n* = 1023)	*p* Value
Age (years)	65 ± 9	72 ± 7*	77 ± 7*^,†^	<0.001
Male (percentage)	786 (76)	737 (72)	737 (72)	0.015
BMI (kg/m^2^)	24.8 ± 3.9	23.9 ± 3.5*	23.3 ± 3.6*^,†^	<0.001
AF type				<0.001
Paroxysmal AF	464 (45)	371 (36)	285 (27)	-
Persistent AF	202 (19)	229 (22)	252 (24)	-
LS-AF	348 (34)	416 (40)	476 (46)	-
Unknown	8 (0.7)	6 (0.5)	10 (0.9)	-
Hypertension	724 (70)	732 (71)	730 (71)	0.92
Diabetes mellitus	235 (23)	224 (21)	236 (23)	0.78
Heart failure	204 (19)	215 (21)	267 (26)	0.002
Vascular disease	116 (11)	126 (12)	136 (13)	0.40
Stroke or TIA	104 (10)	117 (11)	124 (12)	0.36
Major bleeding	11 (1)	6 (0.5)	13 (1)	0.24
AF ablation	153 (15)	71 (7)	43 (4)	<0.001
Antiplatelet use	152 (14)	170 (16)	165 (16)	0.53
NSAID use	22 (2)	19 (1)	10 (1)	0.08
DOAC use	588 (57)	518 (51)	502 (49)	<0.001
Warfarin use	434 (42)	504 (49)	521 (51)	<0.001
CHADS_2_ score	1.5 ± 1.0	1.8 ± 1.1*	2.0 ± 1.1*^,†^	<0.001
CHA_2_DS_2_-VASc score	2.4 ± 1.4	3.0 ± 1.4*	3.4 ± 1.3*^,†^	<0.001
HAS-BLED score	1.2 ± 0.9	1.4 ± 0.8*	1.5 ± 0.7*^,†^	<0.001
Hemoglobin (g/dL)	14.1 ± 1.6	13.7 ± 1.6*	13.3 ± 1.7*^,†^	<0.001
Platelets (×10^3^/μL)	24.4 ± 5.2	19.8 ± 3.3*	15.6 ± 3.3*^,†^	<0.001
BUN (mg/dL)	16.6 ± 7.0	17.9 ± 8.1*	18.8 ± 7.0*^,†^	<0.001
Creatinine (mg/dL)	0.90 ± 0.43	0.95 ± 0.42*	0.95 ± 0.34*	<0.001
CrCl (mL/min)	81 ± 29	64 ± 22*	57 ± 21*^,†^	<0.001
AST (IU/L)	22 ± 7	25 ± 8*	31 ± 18*^,†^	<0.001
ALT (IU/L)	22 ± 7	25 ± 8*	31 ± 18*	<0.001

Values are shown as the mean ± SD or *n* (%). * *p* < 0.05 vs. First tertile, ^†^ *p* < 0.05 vs. Second tertile. LF: liver fibrosis; BMI: body mass index; LS-AF: long-standing persistent AF lasting more than 1 year; TIA: transient ischemic attack; NSAID: non-steroidal anti-inflammatory drug; DOAC: direct oral anticoagulant; CHADS_2_: congestive heart failure, hypertension, age ≥ 75 years, diabetes and stroke; CHA_2_DS_2_-VASc: congestive heart failure, hypertension, age ≥ 75 years, diabetes, stroke, vascular disease, age 65–74 years, and male; HAS-BLED: uncontrolled hypertension (baseline systolic blood pressure >160 mmHg), abnormal renal function (serum creatinine ≥ 2.26 mg/dL)/liver function (chronic hepatic disease [e.g., cirrhosis] or aspartate aminotransferase and/or alanine aminotransferase > 3 times normal range), stroke, prior major bleeding, elderly (age ≥ 65 years), drug use (alcohol/antiplatelet or NSAID); CrCl: creatinine clearance; BUN: blood urea nitrogen; AST: aspartate aminotransferase; ALT: alanine aminotransferase.

**Table 2:** Determinants of stroke and major bleeding events in a multivariate Cox regression model.

Item	Stroke		Major Bleeding	
	HR (95% C.I.)	*p* Value	HR (95% C.I.)	*p* Value
Age ≥ 75 years	1.36 (0.88–2.08)	0.15	1.16 (0.76–1.76)	0.48
Male	1.11 (0.71–1.80)	0.63	1.33 (0.83–2.19)	0.23
Body weight ≤ 50 kg	1.11 (0.63–1.89)	0.70	0.96 (0.53–1.69)	0.91
Persistent AF or LS-AF vs. Paroxysmal AF	1.46 (0.97–2.24)	0.069	1.10 (0.74–1.66)	0.60
Hypertension	1.28 (0.83–2.03)	0.26	1.29 (0.84–2.04)	0.24
Diabetes mellitus	1.05 (0.67–1.61)	0.79	1.31 (0.86–1.95)	0.19
Heart failure	0.82 (0.52–1.32)	0.43	0.79 (0.49–1.24)	0.33
Vascular disease	1.02 (0.57–1.83)	0.92	1.11 (0.62–1.91)	0.69
History of stroke or TIA	2.35 (1.52–3.63)	<0.001	1.01 (0.56–1.69)	0.97
New OAC use	1.71 (1.08–2.70)	0.021	1.28 (0.78–2.05)	0.31
Antiplatelet use	1.00 (0.59–1.69)	0.97	1.18 (0.70–1.94)	0.50
DOAC vs. warfarin	1.06 (0.70–1.58)	0.77	0.97 (0.65–1.45)	0.91
CrCl ≤ 50 mL/min	1.68 (1.06–2.64)	0.024	1.93 (1.23–3.02)	0.004
Third tertile of FIB4 index	1.21 (0.81–1.80)	0.33	1.31 (0.88–1.94)	0.17

HR: hazard ratio; C.I.: confidence interval; FIB4: Fibrosis-4; Other abbreviations were previously defined in Table 1 footnote.

**Table 3:** Determinants of cardiovascular events and all-cause mortality in a multivariate Cox regression model.

Item	Cardiovascular events		All-cause Mortality	
	HR (95% C.I.)	*p* value	HR (95% C.I.)	*p* value
Age ≥ 75 years	1.23 (0.92–1.66)	0.15	2.46 (1.72–3.57)	<0.001
Male	0.82 (0.59–1.13)	0.24	1.96 (1.34–2.91)	<0.001
Body weight ≤ 50 kg	1.23 (0.85–1.77)	0.26	1.68 (1.13–2.47)	0.009
Persistent AF or LS-AF vs. Paroxysmal AF	0.71 (0.54–0.93)	0.015	1.26 (0.91–1.79)	0.15
Hypertension	1.11 (0.84–1.50)	0.44	0.70 (0.51–0.96)	0.028
Diabetes mellitus	1.06 (0.78–1.42)	0.67	1.25 (0.89–1.74)	0.18
Heart failure	1.93 (1.46–2.54)	<0.001	1.46 (1.07–2.00)	0.017
Vascular disease	1.80 (1.27–2.55)	<0.001	1.46 (0.96–2.18)	0.072
History of stroke or TIA	0.81 (0.52–1.23)	0.32	0.97 (0.61–1.48)	0.91
New OAC use	1.80 (1.32–2.47)	<0.001	1.18 (0.79–1.74)	0.40
Antiplatelet use	1.34 (0.95–1.89)	0.086	1.13 (0.75–1.67)	0.52
DOAC vs. warfarin	0.93 (0.71–1.23)	0.63	1.01 (0.74–1.39)	0.91
CrCl ≤ 50 mL/min	1.74 (1.27–2.38)	<0.001	2.17 (1.53–3.09)	<0.001
Third tertile of FIB4 index	1.72 (1.31–2.25)	<0.001	1.43 (1.06–1.95)	0.019

Abbreviations were previously defined in the footnotes of Table 1 and Table 2.

**Table 4:** Evaluation of increased predictive ability of the Fibrosis-4 index to CHA2DS2-VASc score for prediction of cardiovascular events and all-cause mortality.

Risk score	C-statistics (95% C.I.)	*p* Value	NRI (95% C.I.)	*p* Value	IDI (95% C.I.)	*p* Value
*Cardiovascular events*						
CHA_2_DS_2_-VASc score	0.609 (0.574–0.644)	Ref.		Ref.		Ref.
CHA_2_DS_2_-VASc score+ FIB4 index	0.638 (0.602–0.674)	0.025	0.32 (0.19–0.45)	<0.001	0.010 (0.005–0.015)	<0.001
*All-cause mortality*						
CHA_2_DS_2_-VASc score	0.626 (0.587–0.664)	Ref.		Ref.		Ref.
CHA_2_DS_2_-VASc score+ FIB4 index	0.672 (0.636–0.709)	0.001	0.40 (0.25–0.54)	<0.001	0.011 (0.005–0.018)	<0.001

NRI: net reclassification improvement; IDI: integrated discrimination improvement; Other abbreviations were previously defined in Table 1 footnote.

## Appendix A

The key personnel and institutions participating in this registry are as follows: Chief investigator: Hirayama, A. (Division of Cardiology, Department of Medicine, Nihon University School of Medicine); Vice-chief investigator: Okumura, Y. (Division of Cardiology, Department of Medicine, Nihon University School of Medicine); Steering Committee: Kunimoto, S.; Okumura, Y.; Kato, M. (Division of Cardiology, Department of Medicine, Nihon University School of Medicine); and Statistical analysis: Udagawa, S. (Department of Mathematics, Nihon University School of Medicine). Participating institutions: Division of Cardiology, Department of Medicine, Nihon University School of Medicine (Hirayama, A.; Okumura, Y.; Watanabe, I.; Hiro, T.; Takayama, T.; Kunimoto, S.; Nakai, T.; Kato, M.); Department of Cardiology, Nihon University Hospital (Yokoyama, K.; Matsumoto, N.); Kawaguchi Municipal Medical Center (Tachibana, E.; Kuronuma, K.); Yokohama Chuo Hospital (Oiwa, K.; Matsumoto, M.); Sekishindo Hospital (Kojima, T.); Asaka Medical Center (Haruta, H.); Tokyo Rinkai Hospital (Nomoto, K.; Sonoda, K.); Kasukabe Medical Center (Arima, K.; Kogawa, R.); Yasuda Hospital (Takahashi, F.); Itou Cardiovascular Clinic (Itou, T.); Makita General Hospital (Kotani, T.); Itabashi Medical Association Hospital (Okubo, K.); Kondo Clinic (Kondo, K.); Ukima Central Hospital (Fukushima, S.); Keiai Clinic (Chiku, M.); Ohno Medical Clinic (Ohno, Y.); Onikura Clinic (Onikura, M.); Kobari General Clinic (Kobari, C.); Chikuma Central Hospital (Tokai, K.); Kondo Clinic (Kondo, K.); Yamada Clinic (Osamura, Y.); Higashi Saitama General Hospital (Fukuda, Y.; Nakahara, S.); Zengyodanchi Ishikawa Clinic (Ishikawa, N.); Tokutake Clinic (Tokutake, E.); Sugino Clinic (Sugino, K.); Yokohama Sotetsu Bldg. Clinic (Mori, H.); Suzuki Clinic (Suzuki, Y.); Fujita Clinic (Fujita, M.); Yumikura Clinic (Yumikura, S.); Keiai Hospital (Ando, H.); Sekimachi Medical Clinic (Shin, I.); Tokiwadai Surgical Hospital (Mochizuki, R.); Hikarigaoka Clinic (Tokuyasu, Y.); Osuga Clinic (Osuga, E.); Nakai Clinic (Nakai, K.); Kurumatani Clinic (Kurumatani, H.); Horinaka Hospital (Sato, C.); Nasu Red Cross Hospital (Akabane, M.); Sato Clinic (Sato, K.); Kofune Clinic (Kofune, T.); Ikebukuro Okubo Clinic (Yamane, A.); Minami Machida Hospital (Horie, T.); Ishii Clinic (Ishii, N.); Akabane Central Hospital (Ominato, M.); Hirose Clinic (Hirose, S.); Sekimoto Memorial Hospital (Sekimoto, M.); Matsumura Clinic (Matsumura, K.); Tanaka Clinic (Tanaka, M.); Nakadai Clinic (Ogawa, T.); Naruse Clinic (Naruse, K.); Narimune Clinic (Kato, A.); Miyata Clinic (Miyata, H.); Aoyama Clinic (Aoyama, H.); Hara Clinic (Hara, K.); Yano Clinic (Yano, F.); Sekino Hospital (Sekino, H.); Kurokawa Clinic (Kurokawa, H.); Ishii Clinic (Ishii, T.); Abe Clinic (Abe, Y.); Kikuchi Clinic (Kikuchi, T.); Imamoto Clinic (Imamoto, S.); Nirenoki Clinic (Nagai, C.); Kachidoki View Tower Clinic (Sugino, K.).
